# First impressions matter: Mundane obstacles to a forensic device for probabilistic reporting in fingerprint analysis

**DOI:** 10.1177/03063127251333074

**Published:** 2025-05-07

**Authors:** Simon A Cole, Justin L Sola

**Affiliations:** 1 Department of Criminology, Law & Society, University of California, Irvine, CA, USA; 2 Sociology & School of Data Science and Society, University of North Carolina, Chapel Hill, NC, USA

**Keywords:** statistics, forensic, mundane, fingerprint, market, software, device

## Abstract

This article investigates why statistical reasoning has had little impact on the practice of friction ridge (or ‘fingerprint’) examination, despite both interest and some modest scientific progress toward this goal. Previous research has attributed this lack of results to practitioner resistance and legal apathy. This article seeks to complement those explanations through interviews with experts with a variety of perspectives on contemporary fingerprint practice about practical and mundane obstacles to the belated statistical revolution in fingerprinting. Based on these interviews, we argue that a ‘forensic device’ is required to incorporate statistical reasoning into fingerprint practice. This device would consist of a robust statistical model fronted by accessible, usable software. These components, in turn, require other components, such as large research data sets, markets, early adopters, government clients, education, and training. We conclude that the statistical revolution has been delayed not just by grand debates over the probabilistic nature of fingerprint evidence, but also by the seemingly mundane problems posed by developing and maintaining the kind of forensic device that would make such a revolution possible and practical.

Over the past three decades, the field of forensic science has been riven by controversies. Often, these controversies have been framed by epistemological issues central to science and technology studies (STS), such as what authorizes particular knowledge claims as ‘scientific’.

One contentious issue is commonly called the ‘reporting’ of the results of forensic analysis. Historically, little attention was paid to reporting, and forensic analysts often resorted to folksy vernacular terms like ‘match’, especially when testifying in the non-scientific space of the courtroom. As criticisms of such practices mounted, an intervention was engineered by the scientific discipline known as statistics. It was argued that the results of forensic analysis should, or even must, be reported in a probabilistic manner—which is not necessarily equivalent to reporting in quantitative manner. To do otherwise was to defy logic (e.g., Aitken & Taroni, 2004; Robertson et al., 2016).

As sociologists of science, we are predisposed to treat such aggressively totalizing claims with skepticism. But what we might call the ‘statistical turn’ in forensic science raises interesting issues for STS. Of particular interest is the juxtaposition of forensic statistics’ near-total dominance in the scholarly community with its struggles for impact on practice. Despite an avalanche of academic output, government and industry committees, and blue-ribbon reports, the statistical turn in forensic science has yet to fully materialize, with the notable exception of DNA analysis. This is especially true of the U.S., compared to regions and countries where statistical reporting has made more progress, such as Europe and New Zealand (Dutton, 2017).

Much has already been written about why probabilistic reporting is rarely used in contemporary forensic practice outside of DNA analysis. Most accounts have focused on two factors: practitioner resistance (e.g., Abraham et al., 2013, p. 132; Morrison, 2022, p. 5; Swofford & Champod, 2021, p. 1; Swofford, Cole, & King, 2020) and legal apathy (e.g., Cole, 2009, p. 249; Mnookin, 2009, p. 1243).

Arguments can be made for both explanations. Practitioners have been skeptical of—and often hostile to—statistical approaches, exhibiting ‘algorithm aversion’ (Swofford & Champod, 2021, p. 4). Courts wield unique leverage over the situation as the primary ‘customers’—a term increasingly used without irony in forensic science—of forensic science services. They have shown themselves to be unconvinced by forensic statisticians’ announcement of a new way of interpreting evidence and unwilling to demand that evidence be presented in the manner that forensic statisticians insist is the only proper way to present it. These two forces, it is argued, have created a double whammy in which practitioners are reluctant to change their practice and courts disincentivize them from doing so, a situation that has provoked much critical commentary.

However, we suggest here that these explanations may have attracted disproportionate attention in academic journals, media accounts, and the various organizations and commissions engaged in efforts to improve forensic science. One reason for this disproportionate attention may be that practitioners have engaged in epistemic ‘resistance’ to forensic statistics. The debate over statistical reporting invokes philosophy of science concerns about the fundamentally probabilistic nature of the universe, the counterintuitive mysteries of Bayesian probability, and the question of whether ‘science’ is fundamentally a search for certainty or (as most philosophers and statisticians would have it) an exercise in the measurement of uncertainty.

But does the grand debate over probability fully explain the absence of such a model? We ourselves have done research exploring practitioner resistance and legal apathy (Swofford, Cole, & King, 2020) and the present study seeks to complement this work by highlighting other obstacles that may help explain the failure of probabilistic reporting. The explanation might as easily lie in ‘business models’, ‘value propositions’, software support and updates, informed consent procedures for obtaining data, and training programs. These are mundane obstacles (e.g., Gardner & Neuman, 2023, p. 205), rather than epistemic debates (self-styled as grand) between practitioners, lawyers, and statisticians about the fundamentally probabilistic nature of the universe. These issues invoke a different, but equally central, array of STS issues that are more technological than epistemic. They include the process of invention and innovation, how technological dissemination occurs, the open-source software movement, and the role of algorithms in society.

In this study, we set aside practitioner resistance and legal intransigence and explore the more mundane obstacles to the development of statistical forensic devices. This article shares the premise of Swofford and Champod’s (2021, p. 2) paper on forensic algorithms that ‘challenges to implementation are unlikely to be solved by improvements to technology or the mere proliferation of the tools alone’. While Swofford and Champod investigate how to achieve practitioner acceptance, our investigation focuses on the obstacles to getting those tools into users’ hands.

## STS and forensic science

There has been much recent attention in STS research on forensic science. Generally speaking, this work has focused more on epistemological questions than technological ones: such as how traces become evidence and associated legal disputes over evidence (e.g., Granja & Machado, 2023; Jong, 2023; Kruse, 2016; M’charek, 2008; Toom, 2012).

STS scholars have paid less attention to the development of technologies and innovation in forensic science, despite these being central concerns of STS in other domains (e.g., Bijker, 1995; Bijker et al., 1987). Our framework was inspired by Ellebrecht and Lipphardt (2021) who called for more attention to what they termed ‘forensic devices’ in mediating between forensic research, practice, and markets. They proposed this concept because, they argued, ‘while the relationship between forensics and law is already well studied, the intersections of forensics and the society/public, forensics and basic science, and forensics and the police have hardly been explored so far.’ They ‘propose[d] to take a closer look at these relations by using the example of forensic devices. By forensic devices’, they clarified, ‘we do not only mean machines and tools, but also databases, information technologies, and procedures guiding forensic practices.’ We found this notion useful because it drew our attention away from epistemological debates and toward the material existence or non-existence of technologies.

The STS literature on forensic science has not focused on the development of such devices. Lynch et al. (2008) discuss various ‘fixes’ for early DNA profiling techniques, but these are not conceptualized as devices. Rees (2015) studied the colposcope, a forensic device, but he was concerned with the impact of the device on medico-legal knowledge and disputes, not with the development of the device itself. STS analyses of lie detection machines (e.g., Alder, 2007; Balmer, 2015, 2018; Bunn, 2012; Littlefield, 2011; Lokaneeta, 2020), and perhaps other surveillance technologies (e.g., Guzik, 2016), study the development of devices, but these devices are perhaps barely mainstream ‘forensic science’. The STS contributions that come closest to what Ellebrecht and Lipphardt call for is C. J. Lawless and Williams’ (2010) study of Case Assessment and Interpretation (CAI) by the United Kingdom Forensic Science Service (FSS), and more generally C. Lawless’ (2017) studies of forensic innovation. While CAI, a ‘technology of forensic reasoning’ in C. J. Lawless and Williams’ (2010) words, was not a physical device, it fits Ellebrecht and Lipphardt’s broad definition of a ‘device’. Moreover, Pullen-Blasnik et al.’s (2023) recent study of probabilistic genotyping, draws, like ours, on the same notion of a forensic device in arguing that it destabilizes the authority of human experts.

Social scientists have produced a substantial body of work on algorithms, including the ethical issues they raise and their embedding and ‘washing’ of human biases into purportedly objective decision-making tools. These technologies advance false claims to ‘objectivity’ and freedom from discrimination, using their mechanical nature to obscure the traces of bias inscribed into their programming (e.g., Benjamin, 2019, p. 3; Eubanks, 2017, pp. 127–173; Muñiz, 2022, pp. 77–94; Noble, 2018, p. 1). ‘Algorithms are presented as more rational and objective than “gut feelings” or discretionary judgments.’ (Brayne & Christin, 2021, p. 609) Many of those concerns are relevant here. As with other algorithms, our forensic device has been promoted as an ‘objective’ solution to the problem of subjective bias in human decision-making. Most scholarly critiques of algorithms concern ‘bias’ in the sense of discrimination against stigmatized groups of people. In forensic science, the term ‘bias’ is typically used in a more technical sense to refer to cognitive process that skew decision-makers toward a particular outcome, rather than bias against stigmatized groups, although some scholars have argued for a connection between the two (Dror et al., 2021; Puracal & Kaplan, 2022). Thus, whereas predictive policing or risk assessment algorithms, for example, purport to replace discriminatory human judgments with objective machine judgments, forensic algorithms purport to replace human judgments vulnerable to unconscious cognitive biases with machine judgments impervious to such effects (Benjamin, 2019, p. 3).

A forensic device, as we describe it here, could be applied to a range of evidence including fingerprints, firearms and toolmarks, footwear and tire marks, and handwriting. We decided to narrow our scope to a single discipline, selecting friction ridge (‘fingerprint’) examination for our study for a variety of reasons. Fingerprint examination remains a robust and widely used forensic service, even in an age increasingly dominated by DNA profiling, with a relatively large cadre of practitioners and reasonable number of associated vendors, researchers, and so on. It is among the oldest of the surviving forensic disciplines, and is also among the non-DNA disciplines in which the efforts toward a statistical device have progressed the furthest. And, although there continues to be significant resistance to probabilistic approach in the fingerprint discipline, there also appears to be some openness to such approaches (Swofford, Cole, & King, 2020).

In this article, we conceptualize something commonly described as ‘a statistical model for fingerprint associations’ as a ‘forensic device’ in the sense described by Ellebrecht and Lipphardt. A statistical model is, of course, a conceptual tool, an algorithm that might exist in computer code or a scientific publication. But the desired forensic device is more than that. It would also consist of the software interface and workflow that would enable this model to be *used* by the thousands of forensic service providers around the world that perform friction ridge examinations. Thus, the device we are describing is not merely a statistical model nor just a software program but rather a statistical model embedded in software.

## Context for a forensic device

Forensic science has been ‘controversial’ for at least two decades, if not longer (e.g., Pyrek, 2007). A variety of criticisms have been made and reforms proposed. However, the most persistent, prevalent, and programmatic efforts at reform, has come in the form of what can, without irony or cliché, reasonably be called a scientific ‘paradigm’ (Kuhn, 1962) known as ‘forensic statistics’ that has brought about what we might refer to as a ‘statistical turn’ in forensic science, pertaining to all forensic science.

Fingerprint examination seeks to assess whether two or more ‘impressions’ of ‘friction ridge skin’ (the corrugated skin found on fingers, palms, and soles) may have originated from the same source skin. Examiners do this by assessing whether the friction ridge detail visible in those impressions corresponds. If some visible details disagree, the impressions may be excluded from sharing a common source. If all visible details are in correspondence, the impressions might share a common source.

So far so good. The difficulty arises when seeking to attach meaning to a finding of correspondence. Forensic statistics posit that a finding of correspondence necessarily poses a question about rarity. How common are those features that were found to correspond? The spatial relationship between a very small number of details might be quite common, while the spatial relationship between a very large number of details might be very rare.

For nearly a century, fingerprint examiners addressed this issue by positing an imaginary threshold of a quantity of information at which the rarity of that spatial arrangement dropped to one—only a single area of friction skin in the universe could possess that spatial arrangement. The problem was that this threshold had neither been derived through scientific work nor clearly articulated. The threshold was either arbitrarily quantified (i.e., a number of features was simply made up), or not quantified at all and then based on the examiner’s verbal characterization of their intuitive sense of the rarity of various spatial arrangements of details (Cole, 1999). When the quantity of information surpassed the imaginary threshold, the examiner would report to the factfinder that the person known to have made one of the impressions was also the source of the other impression.

Over the last several decades, this approach came under increasing criticism (e.g., Campbell, 2011; Champod & Evett, 2001), including from STS scholars (Cole, 2001; Mnookin, 2001), and was defended by ever smaller portions of the fingerprint discipline. As recounted by Lynch et al. (2008) and others, the development of DNA profiling fueled this criticism because DNA scientists were able to generate defensible estimates of the rarity of particular DNA profiles. For simple DNA samples (i.e., those containing large quantities of genetic material from no more than two ‘contributors’), these rarity estimates were quite straightforward to calculate. This was because simple DNA profiles consisted of rather simple data_—_essentially strings of whole numbers that needed to be multiplied together. DNA scientists also had datasets that had been statistically sampled and more information about the statistical independence of different features. The comparable data for fingerprint comparison was more complex: essentially images of unruly patterns, with little statistical sampling and little known about the statistical independence of the features. And most other forensic disciplines (firearms, handwriting, shoe prints, tire prints, etc.) were more like fingerprints than they were like DNA.

A forensic device would offer a solution to this dilemma by generating a defensible estimate of the rarity of any given spatial arrangement. This would enable the examiner to report their findings in the likelihood ratio format preferred by forensic statisticians—that is, as the probability of the finding of correspondence if the impressions derive from the same source over the probability of the finding of correspondence if the impressions derive from different sources (e.g., Aitken & Taroni, 2004; Robertson et al., 2016). Forensic statisticians and others argued that such a report would be scientifically defensible in a way that traditional fingerprint reports (e.g., ‘this skin is the source of this impression’) were not (Champod & Evett, 2001).

However, as noted above, because fingerprint impressions are visual patterns, rather than whole numbers, a statistical model for fingerprints is mathematically challenging. For most of the 20th century, statistical work on fingerprints focused on estimating the probability of two duplicate fingerprint patterns existing, which is not quite the same as estimating the rarity of a given fingerprint pattern (Stoney & Thornton, 1986).

To be sure, practitioners have reasonable concerns about the use of algorithms, such as lay comprehension of their results and the underselling or overselling of evidence (Swofford, Cole, & King, 2020). However, as Zarsky (2016, p. 122) notes, when challenging algorithms, it is important to ask ‘what are the alternatives?’ because ‘Humans tend to make systematic and predictable mistakes, and our decisions are subject to bias.’ In the case of our forensic device, the alternative depends on whether one accepts forensic statisticians’ postulate that the rarity of observed features *must* be stated when evidence is reported. If one does *not* accept that premise, then the alternative is that examiners may simply offer conclusory opinions like ‘identification’. If, on the other hand, one *does* accept the premise, then the alternative can only be that examiners make up rarities based on their training and experience.

## Automated fingerprint identification systems

For most of the 20th century, fingerprint impressions of unknown origin were either (1) compared to the fingerprints of persons of interest identified through other means (i.e., suspects, victims, etc.), or (2) ‘manually’ searched in databases of fingerprint cards containing all 10 fingerprints of people whose fingerprints had been recorded by the state (i.e., people with criminal records). These ‘manual’ searches were aided by a system of ‘fingerprint pattern classification’ by which the examiner might be able to use the ‘pattern type’ of the unknown impression(s) to narrow in on a subset of candidates from a large database. Despite this aid, manual searches were time consuming and laborious.

In the mid-1980s, technological advances enabled the marketing of commercial computer tools for automating searches of fingerprint databases. These tools, and their attached databases, became known as Automated fingerprint identification systems (AFIS).^1^ AFIS are search aids, not ‘identifiers’. No vendor has ever marketed an AFIS claiming that it is able to determine the source of the kind of partial fingerprint impression that might be recovered from a crime scene. Rather, AFIS generate lists of candidate sources for human examiners to consider, much in the way that internet search engines generate candidate web pages in response to user queries.

### The forensic science service (FSS)

In the 1990s, the FSS, the leading forensic organization in the UK, which had ‘a significant R&D capacity’ (C. Lawless, 2017, p. 391), began a research and development effort to develop the kind of forensic device we are discussing. By the early 2000s, the FSS had begun marketing efforts, and Cole saw an actual sales brochure for the product around that time; the device seemed imminent.

However, in a sudden turn of events, the FSS closed, and the device disappeared. Several years earlier, the FSS, which had been run by the Home Office, was privatized as part of the broader privatization of government services, and it had been expected to become a self-funded enterprise (C. J. Lawless, 2011). The fingerprint statistical model may well have been perceived as a potential source of revenue. Within a few years, however, the FSS collapsed under the weight of its own budget, leaving the UK without a nationwide forensic service provider and leaving the world without the device. As discussed below, an ‘intellectual property nightmare’ would prevent the device’s developers from marketing it themselves after the closing of the FSS. The demise of the well-respected FSS was seen by many in the forensic community as a tragedy and a demonstration that quality forensic science requires government investment not just market forces (Budowle et al., 2011; C. Lawless, 2017, p. 392).

The underlying statistical work behind the FSS model was eventually published by Neumann et al. (2012), accompanied by numerous commentaries, some of them suggesting technical flaws in the model. Neumann would later accept some of these criticisms (Hendricks et al., 2021). While the publication demonstrated that a statistical model for fingerprint was possible, the ‘model’ existed only on the pages of scientific journal. There was no software, no tool, no device. The model was never used in a criminal investigation or court case.

### The ‘Swiss model’

Around the same time, researchers from the University of Lausanne released a statistical model that some call ‘the Swiss model’. Unlike the FSS model, the Swiss model had an associated software interface. It was, however, software for sophisticated computer users, not ordinary laypeople or even many forensic practitioners.

The Swiss model was available for free (to those sophisticated enough to use it). But there was no software company and no regular technical support other than the academic researchers who had first developed it. One obtained it by writing directly to those researchers. As such, the Swiss model was generally characterized in the forensic community as an academic project that was impractical for use by a forensic service provider. It was simply too informal.

### FRStat

In the 2010s, the Defense Forensic Science Center (DFSC), the primary forensic service provider to the United States military, made available a statistical tool called ‘FRStat’ (Swofford et al., 2018). The DFSC began using the tool and testified about it in cases. The mathematics behind FRStat did not appear to be as sophisticated as that behind the FSS model. But FRStat, crucially, had a software interface. Moreover, the DFSC made FRStat available for free to anyone who requested it. If the FSS publication showed that a statistical model could exist in the abstract, FRStat showed that one could exist on anyone’s computer: a forensic device. How good or sophisticated a forensic device was not yet clear. FRStat generated a great deal of discussion, resistance, and criticism. After a few years, a sustained technical criticism of FRStat was published (Neumann, 2020; Swofford, Zemp, et al., 2020).

### Probabilistic genotyping

As these developments progressed, events in the world of DNA profiling continued to loom over debates about a forensic device for fingerprints. By the 21st century, technologies for DNA profiling had become so sensitive that forensic service providers were increasingly faced with what were called ‘complex mixtures’ of DNA, rather than the ‘simple’ samples described above. Complex mixtures were characterized by smaller amounts of genetic material, larger and uncertain numbers of contributors, or both these factors in combination (C. J. Lawless, 2012; Pullen-Blasnik et al., 2023).

The complexity of these mixtures transformed the estimation of the rarity of the findings, once a simple problem of multiplying whole numbers, into a statistical nightmare. Multiple contingencies had to be considered and accounted for simultaneously. Did the mixture consist of four contributors, or five, or six? Each might be possible, but each was not equally likely. Was a particular finding better explained as ‘noise’ or as a valid representation of very small DNA contribution? Statistical workarounds were developed, but these were criticized as flawed. Worse, they could be biased if they estimated a rarity *after* the analyst had committed to a single interpretation of the evidence, rather than taking into account all the possible interpretations of the evidence (Langford, 2015; Pullen-Blasnik et al., 2023; Thompson, 2009).

The solution to both problems—statistical complexity and bias—was a computer algorithm, and tools for calculating likelihood ratios for complex mixture began to emerge in the 2000s (Coble & Bright, 2019, p. 222; Kirchner, 2016). There was robust interest in open-source approaches, and a number of open-source products were released (Coble & Bright, 2019, p. 223; Gill & Haned, 2013; Haned, 2011; Haned et al., 2016, p. 107), but technical support was a concern. Eventually, two commercial products became most prominent. The first, TrueAllele, had a more aggressively entrepreneurial free-market feel. The second, STRMix, had a more government-industry hybrid feel in that it had been supported at various times by the New Zealand government and the United States National Institute of Standards and Technology (NIST).

Without going into excessive detail, probabilistic genotyping did little to persuade the fingerprint community that the existence of a forensic device would usher in a period of peace and harmony. TrueAllele and STRMix became bitter rivals, attacking one another and perceived critics in court, in the media, and in the discipline and accusing one another of various transgressions. Sometimes they yielded very different results when analyzing the same evidence (Coble & Bright, 2019, p. 222; Stiffelman, 2019, p. 124; Thompson, 2023). As the results of probabilistic genotyping began to be used against criminal defendants, those defendants demanded access for their own experts to the ‘source code’ of the products, complaining about ‘algorithmic secrecy’ (Brayne, 2021, p. 86). The vendors argued that their source code was proprietary, and extensive legal battles ensued and continue (Cino, 2018; Eidelman, 2018; Imwinkelried, 2016; Moss, 2015; Roth, 2017; Wexler, 2015).

## The present study

The present study was undertaken under the auspices of a large, multi-university, government-funded center of excellence in the U.S. The authors were co-investigator and researcher of the Center for Statistics and Applications in Forensic Evidence (CSAFE), whose goal is to develop statistical knowledge and tools for forensic science. While statisticians were at the academic core of CSAFE, social scientists were included as well with a mission to study the implementation of the tools developed by the statisticians.

CSAFE was funded in the aftermath of a dramatic event in U.S. (and global) forensic science: the publication of a report by the U.S. National Research Council (NRC, 2009) strongly criticizing the state of forensic science. Though the report concerned the U.S. only, it attracted attention around the world. While some perceived the NRC’s criticisms to apply to forensic science everywhere, others perceived the report as parochial and oblivious to developments in other regions (e.g., Margot, 2010). Although forensic practice is, of course, highly globalized, its development and implementation have also varied greatly between countries (Julian et al., 2022, p. 25).

The NRC’s primary recommendation was the creation of a new independent government agency to regulate and promote forensic science. The NRC explicitly specified that this agency should be separate from the U.S. Department of Justice, based on the reasoning that the embedding of forensic science in law enforcement, rather than scientific institutions, was at the root of many of its ills. The NRC suggested the National Institute of Standards and Technology (NIST) as a science-based government agency with a history of contributions to forensic science that could be helpful in efforts to bring more science to forensic science. The NRC’s imagined new government agency did not come to pass, but a number of less ambitious interventions did. Some of these were driven by NIST, including funding for a center of excellence in forensic statistics that was eventually awarded to the coalition that became CSAFE.

That statistics was perceived as the scientific discipline that was needed to improve forensic science, was itself an intriguing choice. The utility of statistics to forensic science lies primarily in its promise to help interpret evidence, rather than, for example, developing new detection or analysis tools. The funding of a center of excellence in statistics reveals the extent to which poor or misleading interpretation of evidence was perceived as a key weakness of forensic science. As discussed above, forensic statistics had for years been claiming that it was the solution to this problem. Although the movement had struggled for influence everywhere, it had found its most receptive audiences in Europe and Oceania. U.S. practitioners and courts were perceived as especially hostile to forensic statistics.

Among the mandates for the social scientists of CSAFE was helping statisticians understand how to implement the tools they built into forensic practice. To some extent, this inquiry took the form of understanding why those tools had not yet been implemented. As noted above, we made an initial foray in this inquiry that focused on the ‘prime suspect’: practitioner resistance (Swofford, Cole, & King, 2020). The present study is a sequel.

In contrast to the earlier study, which included a broad survey of fingerprint practitioners, the present study deliberately targeted individuals who we thought would be able to inform our research question. Necessarily, then, our respondents were not a representative sample of any community. Although our respondents had diverse perspectives and opinions, they were all generally sympathetic to the premise that the fingerprint evidence is probabilistic in nature and that statistical tools should, at least in principle, be useful.

We interviewed stakeholders with a variety of perspectives and strong opinions on the obstacles to a forensic device. Our respondents worked for a variety of employers: forensic laboratories, universities, AFIS vendors, government agencies, and private research companies.^2^ Three quarters of our respondents were sampled purposively by us, and one quarter were ‘snowballed’ from our original respondents. Most of our interview requests were honored. After an initial round of interviews, we were disappointed in the number of respondents with expertise in AFIS, and we made a concerted effort to recruit more. We completed 19 interviews with a total of 21 respondents. Two respondents were interviewed together, and one respondent preferred to provide brief answers in writing. Although the bulk of our interview respondents were based in the U.S., six were based in Europe. As noted above, in the U.S. forensic statistics is often seen as a European import, and, although our study was about the U.S., snowball sampling inevitably led to suggestions for European interview respondents.

The interviews were semi-structured; we had a short list of questions, but the discussions ranged widely from our original questions. Interviews ranged in length from 18 minutes to more than two hours, with a median length of 38 minutes. The first interview was conducted by Cole, the remaining interviews were conducted by Sola.

The identities of the authors and the study’s affiliation with CSAFE were known to the respondents. Cole has published research that is generally supportive of probabilistic approaches to forensic evidence (e.g., Cole, 2009), while stopping short of some of the totalizing claims, such as the claim that it is the only logical approach. As we discuss below, though, supporting probabilistic reporting does not necessarily imply supporting everything that will be needed for a forensic device. Respondents were aware of his participation, and he was occasionally referred to in the interviews.

Respondents were told the interviews would be recorded. We have kept all respondents anonymous. Where necessary, we have edited quotations to remove identifying information such as names of corporations, organizations, or countries. Because English was not the first language of all respondents, we also lightly edited quotations for consistency with standard English to preserve anonymity.

We analyzed the data thematically using Atlas.ti. In our first pass through the data, we used open-ended coding, arriving at 42 codes. We then aggregated these themes into five general themes: Data, Research Community, Resources, Software, and Innovation Dissemination. We discuss each of these below in an order that makes narrative sense; the order is not intended as a quantitative measure of the themes’ importance. Likewise, we do not attempt to quantify the frequency of the themes within the interview data; we do not think such information would be meaningful given the modest number, semi-structured format, variable length of the interviews, and the fuzziness and arbitrariness of the boundaries between themes. Quotations from the data are followed by the interview number. Interviews are numbered by the order in which they were conducted.

## General themes

### Data

We did not expect the availability of data to be among the issues raised by our respondents. Outsiders might assume that fingerprint data is easy to come by, it is, after all, relatively simple and low-tech to record fingerprint impressions. The process is neither physically invasive nor time-consuming, and government databases contain millions of samples.

However, the data required for research to develop statistical devices for fingerprint examination turns out to be extremely difficult to obtain. Interviewees mentioned easy access to large, useful data sets as an important obstacle to the development of a statistical device. Governments tend not to share their large databases of fingerprints, probably because they are working tools of law enforcement. However, governments are reluctant to share even ‘defunct’ fingerprint data (e.g., from deceased people) because of privacy and consent concerns. In the United States NIST has long made a small database of fingerprints available to researchers. It recently discontinued this database because it decided, retrospectively, that the donors had not been properly consented:

We need data. We need a lot of fingerprints that come from known sources. A lot of the early models were built using the data in this old SD 27, the special database 27, which is problematic. People need to stop using that database. It’s_—_actually the NIST pulled it down. You can’t even get that database anymore, but so many people had already downloaded it that they’re still using it. So, we need new databases that people can draw on to get their samples. (13)

NIST has replaced this database with a new improved database:

It seems there’s a good nucleus to produce a statistical model. It seems the nucleus is there and now we have to grow it. We have to grow it into something bigger. And to me, that’s going to mean a lot of data. I know NIST, they’re developing a dataset now that has close to 100,000 paired images in there. Yeah. And to me, that is number one. You have to have a lot of data. So a huge representative dataset that goes from zero to a 100, as far as quality and quantity and everything like that. (10)

One valuable data source seems to have been the result of a lucky accident of government cooperation:

One reason why we have had the chance to be active in this area is that at some point—but we are talking about 25 years ago some of the [country] government allowed us to use a large amount of their cards for fingerprint research. The availability of millions of fingerprints is a prerequisite to do any research where you want to base your estimates on data. (15)

However, this was seen as a one-off success unlikely to be repeated by any government today:

Since potential research groups will start to say, ‘well, give us some data to play with’, and then the government or agency will say, ‘oh my God, we cannot give you this data because they are protected under Data Protection Act.’ Hence, it makes life a bit difficult. (15)

The FSS model used a database of fingerprints obtained from the FBI, but this data was apparently laborious to obtain and unlikely to be repeated:

Basically, because the data that would be necessary to do it is held by the FBI and they will [not] let anybody see the data. (2)

One way of overcoming privacy concerns is through creating synthetic fingerprint data, (i.e., computer-generated fingerprints [e.g., Engelsma et al., 2015]), but one respondent was skeptical:

research in this field is also difficult because data is scarce. Synthetic data …. Synthetic data doesn’t work. Synthetic data won’t work because if you know the synthetic data …. Because you want to investigate the things that the algorithm cannot do. And when we synthesize data, we synthesize what we know. So what we synthesize is not going to be good for understanding the things that we don’t know. So we have to use operational data. And operational data is really hard to come by. (4)

It may well come as a surprise to outsiders that fingerprint researchers are so starved of fingerprint data that they are seriously weighing the advantages and disadvantages of using computer-generated fingerprints. One researcher has suggested using koala prints to avoid ethical consent issues.

### Research community

Some respondents mentioned the lack of a robust research community around the problem of a statistical device for fingerprints. Our respondents argued that the community of researchers coalesced around the scientific problem of a statistical device for fingerprints is simply too small for normal science to occur:

I think, first of all, in order to build a probabilistic tool in, say, fingerprints, you not only need a good knowledge of statistics, you also need a good knowledge of fingerprints. And the number of people who are at the intersection of the two is relatively small. (6)

This concern is of course, a familiar theme in social studies of science. Under names ranging from ‘paradigms’ to ‘research programs’ to ‘core sets’, STS scholars have argued that scientific problems, if they are to be ‘solved’, require a large enough group of interested and committed researchers (Collins, 1981; Kuhn, 1962; Lakatos, 1978). This community is perceived as necessary for the intellectual ferment that produces scientific innovation. Our respondents told us this ferment is lacking:

I think it will be very important to have a larger community of scientists putting some attention and effort into these fingerprint models. So, I think there is a lack of critical mass when it comes to the number of scientists dealing with these questions. And that leads to very niche, it’s a very niche area. (15)

Our respondents’ comments about the lack of a research community are not surprising considering the relationship between forensic science and universities over the past century. In its 2009 report, the NRC (p. 14) asserted that ‘forensic science and forensic pathology research, education, and training lack strong ties to our research universities and national science assets’. Forensic scientists have long felt neglected by universities and relegated to the realm of ‘applied science’. The relationship between forensic science and universities has ebbed and flowed over the past century, but forensic science has never been truly embedded in universities. Today, it is indisputable that forensic science is only at the margins of the university science establishment (Margot, 2011). There are some forensic science programs at universities, some of which are excellent. But a university does not need a forensic science program to be considered a proper university, and most excellent universities do not have them.

### Resources

A lack of resources was commonly cited as a reason for the lack of progress. Many respondents specifically mentioned AFIS vendors in this discussion. There was a widespread belief that AFIS software, which was highly sophisticated and already used in virtually all identification units, was the logical ‘place’ to locate a statistical model. And yet, no AFIS software had anything like a statistical model built into it.

Many respondents said that an AFIS vendor was the only possible route to creating such a forensic device. Governments, they argued, could not do it:

No government-sponsored initiative is going to be successful; it has to be industry-driven. (8)

But until we have a situation where an AFIS vendor comes onboard, revolutionizes this, makes this a system that is managed and has tech support, and then it’s tested by some kind of grant in which four or five different laboratories independently test that new program, and then the reports are compared, and then there’s a publication that comes out from it, until then, I think that’s the biggest limitation as far as business application. (14)

It’s [AFIS] the ultimate place to be. It’s the only place I see as viable, right? (18)

**Figure 1.**
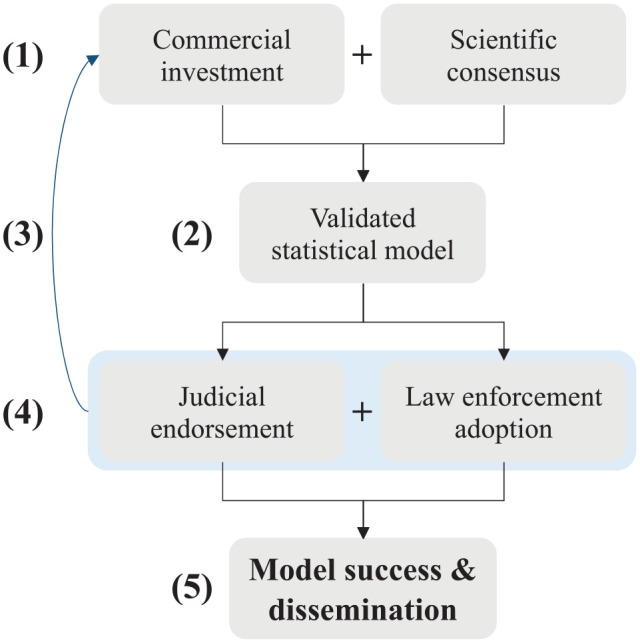
Path dependence of statistical model adoption. Participants believed that **(1)** commercial investment (primarily by AFIS vendors) and scientific consensus was required to develop a **(2)** validated statistical model. However, commercial investment depends upon a **(3)** perception that courts and law enforcement will **(4)** adopt a valid model. Judicial endorsement and law enforcement adoption of a valid statistical model will enable the **(5)** success and dissemination of a statistical model.

Our respondents did not think it was likely that a statistical model would be built into AFIS software in the near future. The primary reason cited for AFIS vendors’ lack of interest in statistical models was the market (Figure 1).

A lot of it has to do with money. It’s ‘follow the money’. Until an AFIS vendor or a big private company gets on board and says, ‘You know what? We see value in this, we’ll build it, and we’ll support it from here to eternity until we stop making AFIS systems’, until they get on board, I don’t believe that they’ll have an accepted model to move forward to. (14)

Indeed, ‘many’ desired forensic innovations ‘are not currently perceived to be commercially viable’ (C. Lawless, 2017, p. 395). More generally, C. J. Lawless (2011, p. 686) has argued that private industry has ‘so far failed to deliver’ a sustainable forensic science system.

It was argued that AFIS vendors did not perceive the addition of a statistical model as a money-making upgrade to their software.

And if there’s no demand then, how can we expect commercial entities to be willing to invest their time and resources to develop something that there’s no demand for. (11)

[T]hey’re going to build the things that they think the most of their clients want and that they can sell. And if it’s not, there’s no bang to the buck, then there’s no incentive for them to put development dollars into it. (13)

It’s a product that does not sell. It’s a little bit just like having a very tricky proficiency test.^3^ (19)

This argument was closely based on another theme: the lack of demand for such a model among the AFIS vendors’ customers. It was argued that customers had little or no interest in statistical models; therefore, why would AFIS vendors invest resources in incorporating one into their software?

One reason it hasn’t reached the market is because there is no market because the customer is not asking for it. (15)

If we have the demand from the practitioner community, like the true demand for it, and they ask their AFIS vendors, ‘Hey, in the next upgrade, we want to have a means of providing a statistical assessment to the match.’ Then I bet they would be willing to consider how to do that. But I don’t think it’s been demanded. They have the capacity to do so. (11)

Indeed, it was argued that customers place higher value on seemingly more superficial software enhancements than a statistical model.

And when suggesting new future developments, new future features, there are definitely other features that are … looked at more positively …. There’s some tools that examiners hear about and go, ‘Yeah, yeah, yeah. You guys build that. That’ll be really helpful for us.’ A stat model is not at the top of the list. (9)

Our respondents expressed a somewhat naïve view of ‘demand’ as something entirely within the control of consumers, rather than something subject to producers’ influence via marketing and investment.

These comments also highlight the extent to which AFIS vendors still orient toward criminal recordkeeping and 10-print identification (i.e., complete sets of intentionally recorded fingerprints), rather than forensic analysis. This may be counterintuitive for some readers because the popular imagination perceives fingerprinting as a tool for crime scene investigation. Arguably, however, its forensic application has always been a ‘side business’ to its primary application in criminal recordkeeping—from its very origins (Cole, 2001, p. 168). Today too, AFIS vendors may well see forensic analysis as a side business to a huge industry maintaining 10-print criminal records and performing automated searches on them.

The example of probabilistic genotyping also loomed over the discussion of AFIS vendors. Some respondents worried that if AFIS vendors were to develop and market proprietary statistical models, they might descend into the same controversies seen in probabilistic genotyping. First, competitive battles between vendors:

When a free market works, great, you now have each one of these vendors throwing hand grenades at each other. They view it like, if we don’t think we’re going to win, we don’t want anyone to win. We want to keep our slice of the pie. (12)

And second, legal battles over the disclosure of proprietary source code:

Well, the problem with the model being made by a commercial entity is that, then the commercial entity has a vested interest in protecting some of the things underlying the hood, such as the specifics about the algorithm itself. And that we’re seeing play out in probabilistic genotyping software. And that’s created its own legal challenges for wider spread adoption as well. Because you have a software from a commercial entity, and no one really knows [what’s] under the hood, how it’s operating. (11)

While most respondents focused on the failure of AFIS vendors to develop a forensic device, one respondent argued that, in fact, AFIS vendors were trying to fashion such a device:

We have done research on statistical models and evaluated how to integrate new AFIS. But again, I personally feel, because that’s actually one of my brainchildren, to really wish, one day, a vision to have a statistical model embedded into AFIS. (18)

Indeed, this respondent was confident that there could be a device within two years of the interview:

[I]t will happen. I will tell you, not just my personal conviction, I think the team is convinced that it needs to happen …. So, for us, it’s a matter of time, to be honest. I wish we had that 10 years ago. That journey started for me 10 years ago, to have this in the AFIS. So, 10 years later, we are not close yet, but we are much closer than ever before. (18)

Still, this respondent too believed market forces were needed:

I think it can only be accelerated if there was a … stronger market attraction. If not, I would say we are going to have to wait another two years. At least two years. (18)

Indeed, this respondent contended that we might have a forensic device now but for another contingent near miss, similar to the near miss by the FSS:

I will give you a back story. I know this is recorded, so I need to be careful, but it’s public knowledge, right? I was with a prior company before I joined [organization], back in 2015. So count that 2013, 2014. I was already working with [name] in exploring a model. I was working with [name] on how to make certain AFIS automated. And we were very close in …. I would say if things [had] gone a certain way and certain things, I think we could have an early version of a tool inside a smaller AFIS before 2015. (18)

The respondent thought that a ‘stronger push back then in 2013, would have something in 2015’. (18). After the completion of our data collection, an AFIS vendor began giving presentations about the incorporation of a statistical model, based on Hendricks et al. (2021), into its software. However, the research is still preliminary and no ‘button’ has been incorporated into the user interface (Ray, 2023).

If market forces will not drive the development of this forensic device, what about other resources? An obvious alternative is government. However, the experience with the FSS did not build confidence in government or quasi-government solutions:

the FSS went out of business, and, again, it became a whole intellectual property nightmare. And essentially the model had to be abandoned. He [Neumann] didn’t have the right to take it out and continue to develop it. So then he went to universities in the States, and he’s been working on new models that are different enough than the old one to be considered a new product. (13)

The government project FRStat did result in a device which is freely available, but it lacks the marketing and technical support that would come with a commercial product:

So FRStat is not currently commercialized at all. So it’s being offered for free. The real obstacles they are partly getting, because it was developed under the DoD [Department of Defense], and we’re dealing with the federal government. It’s getting permissions worked out between DoD and the other agencies who want to use it about who has the right to use it and giving them permissions and all of that. (13)

It was noted that the ‘Swiss model’ was not ‘commercial’ (14)—you literally email someone and ask them to send you the program. More generally, most respondents were skeptical that this could be the solution, primarily because of the need for the mundane infrastructure of computer software, such as technical support, updates, and upgrades. As von Hippel (2017, p. 14, original emphasis) has noted, there is a ‘diffusion shortfall’ in ‘free innovation’ because ‘free innovators may often have too little incentive, from the perspective of social welfare, to invest in *actively* diffusing their free innovations’. One respondent specifically mentioned CSAFE itself as an obstacle to funding:

So I mean, to be honest, I think one of the biggest hurdles in moving forward with this, is the funding to do so, right? And currently, CSAFE has gotten a large amount of money to do work along these lines. And I think when the award was first, granted, there was a lot of … I think, the community had this understanding that CSAFE would move in this direction to develop this. But there hasn’t been the progress that was expected initially. So, as long as I think CSAFE is receiving this funding, I don’t see other funding being directed towards a different group to develop this. (9)

If neither markets nor governments can sufficiently motivate the development of the device, what about a different approach? Some respondents discussed the potential of open-source solutions that might be made freely available, or what von Hippel (2017, p. 1) has called ‘free innovation’. Open-source approaches are appealing in many ways and are often perceived as win-win solutions that have both ethical and technological advantages over market approaches. But those of our respondents who discussed the topic were skeptical:

open-source stuff, but that will never work because once something is open source, then usually there’s very little maintenance, there’s no customer support, there is no upgrades, and so and so on. (8)

Although our respondents did not specifically mention it, they may have been influenced by the experience with probabilistic genotyping and the supposed ‘failure’ of the many open-source solutions that appeared around the 2010s.

Some respondents pointed to other hidden resource needs beyond the mere development of the device, such as validation and training:

So you need staffing. So you need staffing from the research side to develop this thing. Programming, to make sure it’s, to actually create the thing. You need a large amount of testing. You need all the data you can get to throw at this thing to validate it. You need to do that. And I think you also need a pretty robust training program for the end users. You can’t just throw the software at them and expect them to pick it up. They need to really understand what’s going on. (3)

Several respondents noted that the development of a statistical model for fingerprints was a difficult scientific problem:

I think at the highest level, it’s because it’s hard, right. And I don’t think it’s for a lack of trying. (9)

In this regard, fingerprints were contrasted with DNA. Although obtaining access to genetic information (molecules) is more technically challenging than obtaining friction ridge impressions, the statistical modeling for (simple) DNA samples is, counterintuitively, *less* challenging:

The technical reasons I think are more challenging. Fingerprints are a really strange puzzle. You know the information that we rely on is not discrete the way it is in DNA.^4^ (13)

### Software

A forensic device does not consist merely of the scientific work that goes into a developing a statistical model that can withstand scrutiny. For there to be an actual ‘device’, there must be some kind of software that allows line practitioners to access and use this model. It was noted that existing software is in a form that only somewhat computer-savvy users would even be able to run. Most respondents believe that the device requires something much more like the kind of commercial software that is available on most users’ computers:

For it to be successful, the software tool, I think you want an actual software company. We may want that to be commercialized, someone who’s making …. Like I said, you don’t want a Microsoft or Windows update to be able to kill this. You need to have a tool that is owned by a firm that has a real help desk and a real way to troubleshoot so that’s part of it. You want that to be a really user-friendly front end, right? It needs to have a Photoshop …. The complaint with LIM [Laboratory Information Management] systems, right, is you might have one that’s really great as a database, but the user front end is too clicky. Too many things buried in shells where you’ve got to click and open a window and then open a sub window and then get the … You don’t want that. It’s got to be really parsimonious, really straightforward, ideally familiar to examiners. It should feel like the image processing tools that they’re using. That’s part of putting a tool on their hand. (12)

Not only is the currently existing software perceived as not usable, but it also perceived as vulnerable to obsolescence (Slade, 2006). The same respondent noted this in regard to FRStat:

You’ve got to have a latent fingerprint branch that downloads the program and can use it. The first business obstacle is right now, it’s just freeware that Henry Swofford wrote. He’s a pretty smart guy, and it’s pretty good. But it might not survive an update to Windows, right? It might not survive an update to Excel. Henry’s not a software company, right? The first business obstacle is the intellectual property needs to be licensed to someone who’s a software firm in the forensic space who can turn that into a professionally programmed thing that’s robust and will work across software updates and those kinds of things. (12)

Another respondent identified software bugs as another obstacle:

And then when you add software to it, that’s a whole ’nother animal because software doesn’t just run on its own. Software adds another layer of complexity, which needs to be debugged. There are bugs in software that affect the result, and nobody likes to talk about software bugs, but I worked for an AFIS company for [many] years, and I know what bugs are. You need lots of testing to work the bugs out. (10)

### Innovation dissemination

We grouped the final set of obstacles under the broad heading ‘innovation dissemination’. This is a varied group of obstacles that concern a familiar theme in STS: the contingent circumstances that cause some technologies to ‘succeed’ and others to ‘fail’. Our respondents recognized that success begets success—that the device would need to become ‘popular’; it would need to be widely adopted to exert pressure across industry to follow the lead of early adopters. But our respondents identified a number of obstacles to this actually occurring. As one respondent put it:

I think there’s lack of clarity of how the systems should be implemented operationally, and how such implementation will impact existing practices that are established and trusted. (11)

One such obstacle was legal. Although our study was intended to set aside the more common sources of practitioner resistance to probabilistic reporting, such as concerns about how such evidence would be perceived and received in court, our respondents did not always respect our—admittedly fuzzy—boundary, and we were not always able to keep such concerns from leaking into our interviews. Several respondents described a ‘fear’ of using results from such a device in court:

And I can understand that [a] majority of the colleagues around the world are scared about the probabilistic approach. But it would all come down to their fear of how do we present this in court without stuffing something up? And are we equipped in who’s going to train us and who’s going to protect us and what policy protects us if there’s an error? And where does it come from? Who rules it? How do we even begin to implement it? Who does the training? There’s so many things. (7)

Really fear is the main obstacle to adoption. I think that a lot of people are sitting back and waiting for somebody else to go forward. I think that’s [the] main concern. People will know how to use it properly, and explain it properly, and also how it will be taken by the courts. Nobody wants to be the first case to do something and lose the case and set the precedent and have their name associated with that failure. So a lot of labs are … like I said, interested, but they want to see other people do it successfully first. And they are very concerned about training, particularly because many latent print examiners particularly do not have a background in statistics, and they don’t feel comfortable using tools. And then having to explain them to lay people when they don’t even feel that they understand themselves. (13)

This fear is consistent with what Bechky (2020, pp. 75–97) calls a ‘culture of anticipation’ in forensic laboratories centered around the unlikely but intimidating possibility that any individual case ends up requiring an appearance in court. C. Lawless (2017, p. 400) also notes concerns in the forensic community that misunderstanding of novel forensic technologies ‘in court could hold adverse consequences for innovation’.

Another respondent thought the fear was broader and deeper, that probabilistic reporting might fundamentally change the criminal legal system:

And so, the biggest obstacle would be implementing something that would damage the justice system. And I think that’s the biggest fear of people and also probably the biggest risk. (7)

For some respondents, practitioners’ lack of trust and concerns about ‘algorithmic secrecy’ (Brayne, 2021, p. 86) was a key obstacle:

For them, there is a black box. It’s called an algorithm. They don’t have a clue what is used by the algorithm in order to arrive at a specific value. So, there is this inherent mistrust with them …. So, for the statistical model to be implemented in the fingerprint community, you have to have the trust of the people who are doing the reports, and the whole problem with these people or problem, it is that they are accustomed to make this evaluation themselves. (16)

Another seemingly mundane need identified by several respondents was education and training:

And then finally, I think there’s an overwhelming task in front of them to invest in strengthening the practitioners’ fundamental education on these topics, not to mention the training on the specific model that they decide to move forward with implementing. You can train the examiner on the model and how to push the buttons and how to get the result out, but they’re still going to be expected to testify to the interpretation of the results and to the fundamental conceptual operations of the model itself. So that means you have to take this training … a step further and make sure they have the foundational education and basic principles of probabilities, statistics, and logic and inference and so forth. (11)

This concern is borne out by the experience of probabilistic genotyping in which experts have been challenged in their ability to explain results generated by sophisticated software (Pullen-Blasnik et al., 2023).

Several practitioners mentioned a need well known in innovation studies: early adopters (e.g., Galloway, 2011, p. 63) or ‘lead users’ (Oudshoorn & Pinch, 2008, p. 541).

[W]e’re always caught in a chicken and the egg scenario where you want to adopt something, but yet, there’s no early adopters. And the early adopters can’t adopt until there’s a fully working model, and a fully working model can’t be adopted until there’s early adopters. (10)

But who should the early adopters be? Some thought it had to be a large government agency:

So until one of the big agencies, specifically the FBI, some other larger entities, get on board with it, it’s not really going to catch fire because nobody really wants to be that second group to dance with the crazy people like us. (14)

Another respondent cautioned that in the decentralized U.S. criminal legal system, even the leadership of the FBI was not a guarantee of successful dissemination:

Even if the major agencies will move towards this approach, then having the other 18,000 police forces and fingerprint services moving equally to this type of approach in each single criminal justice court all over nation, it’s a complicated matter to have a direct and practical solution. (19)

Others insisted that AFIS vendors could not lead the way alone:

I think it’s also about having a spokesperson, a person that carries out that need, more than just a vendor. Because sometimes when the vendor says something, it’s because of self-interest. You just want to sell your product, right? So when you say, ‘We need a model in the AFIS’, we can only carry that flag for so long until someone else has to say it. Not just some of the progressive experts and researchers, right? Of course they’re going to say, ‘Of course you want to have a model in the AFIS. You are working on a model.’ (18)

The same respondent suggested that some sort of enforcement mechanism, such as an accreditation requirement or an industry standard, was necessary for implementation:

But if someone is not signaling the need, or saying, ‘This is best practice’, or, ‘This has become your certification for accreditation’, there’s many ways to put into a mandate. I just haven’t seen the initiative from all the way to OSAC to see that they want to put that in some way, a mandate. There are so many opportunities that were missed. So, I don’t want to call this out, but OSAC could have put that into the best practice and recommendation.^5^ (18)

Some respondents suggested that FRStat could serve as a ‘bridge’ to the desired forensic device:

FRStat is a bridge to allowing them to see the value of having statistical methods implemented. And then that once the practitioner community sees how a statistical method could fit within the examination scheme, and they start building trust and confidence and comfort around integrating a statistical method, the demand will then theoretically increase. And then you’re going to have the big AFIS vendors and the other commercial entities or, a well-supported … a government entity, whatever it might be, to come along and actually say, ‘Here’s a far more powerful, far more sophisticated algorithm system, that’s going to provide more precise measures of the strength of the fingerprint comparison.’ (11)

One respondent thought the legal system could be a lever for implementation, if it wanted to be:

If you want it to be an easy one, I will give you an easy one. Any testimony to a federal court, the federal agency can say, ‘Without this tool, not admissible.’ (18)

Another respondent seemed to suggest that a scandal would be necessary to bring about implementation:

Traditionally, looking back at history, big crises or big mistakes are a trigger of big changes. (19)

Paradoxically, this informant ended up musing on the possibility of the most mundane of catalysts for change: an error, much in the way that earlier fingerprint errors had provoked significant changes (Cole, 2016).

## Conclusion

**Figure 2.**
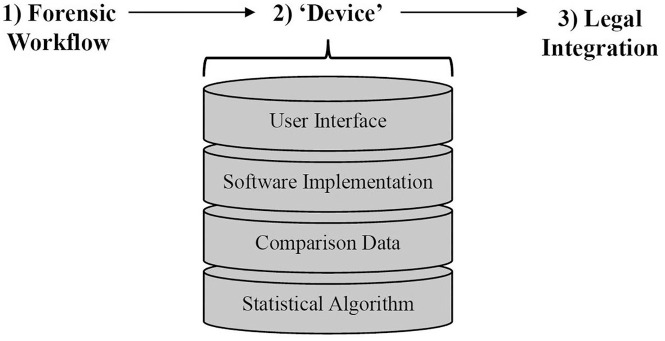
Decomposition of forensic device for probabilistic reporting in fingerprint analysis. Forensic devices are used within the context of **(1)** forensic institution that feature distinct workflows. A **(2)** forensic device for probabilistic reporting in fingerprint analysis must feature several distinct components: a valid statistical model, comparison data that remains valid throughout the lifetime of the device, a reliable software implementation, and a user interface that is amenable to end users. Finally, its reports must be **(3)** amenable to the workflow and sensibilities of legal institutions.

Our interviews show that the realization of a forensic device, even for fingerprint examination—one of the disciplines for which this goal seems most within reach—will require more than many casual observers of forensic science may assume. It will require more than merely educating practitioners to perceive statistics as a tool, rather than a threat. The device itself will require not just a technical solution to the challenging problem of modeling the rarity of fingerprint patterns, but embedding that solution in software that is usable and preferably familiar and integrated with existing software tools already used in practitioners’ workflow. The model itself will require access to large data sets, which remains challenging. The whole device will need to be integrated not only into practitioners’ workflow, but into their occasional forays out of their usual workplaces and into the courtroom. It will likely be necessary to develop a method in advance for integrating statistical claims into the rituals that legal actors use to communicate forensic evidence to factfinders (Figure 2).

In particular, our study clarifies that there is a difference between the abstract desire for probabilistic approaches to forensic evidence and desiring the forensic device that would make those approaches necessary in forensic and legal practice. Our study clarifies that this device would require certain compromises that would, at a minimum, call into question the premise that probabilistic reporting is an unmitigated good. A forensic device would likely include a ‘push-button’ interface that produces impressive numbers through algorithmic processes, which would potentially be challenging to understand and explain, and perhaps obscured by intellectual property claims. It is easy to imagine desiring probabilistic reporting while fearing such a device.

STS as a discipline claims to be a tool for analyzing at once the epistemic and the mundane, a remarkably broad span (Latour, 1992). We see the breadth of this span in the controversy over the attempted intervention by probability into fingerprint analysis. It has now been widely discussed that this intervention has been impeded by practitioner resistance to what we might call epistemological issues—in particular, the attempt to transform the product of fingerprint examination from binary certainty to uneasy gradations of uncertainty.

In this article, however, we follow C. Lawless (2017, p. 390) in arguing that the transition to probabilistic fingerprint interpretation has been slowed not only by ‘perceived differences between forensic practitioners, law enforcement officials and publics over the epistemological status of forensic technology’ but by the ‘role commercial imperatives … may play in complicating these issues’. We have shown that, in addition to epistemological debates, probabilistic fingerprinting is also impeded by seemingly mundane market and technological issues: data, software, resources, user interfaces, training, and so on. Advocates for the transformation of fingerprinting into a fully probabilistic enterprise, then, face the daunting challenge of simultaneously engineering a new epistemology and new technological infrastructure.

Probabilistic fingerprinting cannot yet cross the divide between science demonstration and adopted technology. Technical feasibility is not a major impediment, but technical realization is. In this article we demonstrate that, even if epistemic disagreements were set aside, technological dilemmas of innovation, development, and dissemination serve as obstacles to the development of practical statistical forensic devices.

## Notes

1. Today, it is common to use the broader term Automated Biometric Identification Systems (ABIS). In this article, we use AFIS because that was the term used more often by our respondents.

2. To avoid compromising anonymity we do not provide further detail on the backgrounds of the interview respondents.

3. This is a, perhaps wry, reference to the belief in forensic science circles that many commercial proficiency tests lack rigor as a response to customer desire. Most notoriously, the President of Collaborative Testing Services, the leading commercial provider of forensic proficiency tests in the U.S., candidly told the National Commission on Forensic Science that their tests were deliberately designed to be easy because that was what their customers wanted.

4. The term ‘discrete’ is a reference to the fact that the data structure used in forensic DNA profiling is based on ‘alleles’ which represent the number of times a genetic sequence repeats. The number of repetitions varies between individuals. This allows alleles to be described by whole number values. A sequence cannot repeat a fractional amount—a sequence cannot repeat 1.3 times. Fingerprints lack this unusual, and useful, data structure.

5. OSAC, the Organization of Scientific Area Committees for Forensic Science, is an initiative of the United States National Institute of Standard and Technology to promote standards for forensic science in the U.S.
